# Use of a SimBox, a Video-Augmented, Newborn Resuscitation Simulation for Prehospital Providers to Measure Clinical Performance and Educational Experience

**DOI:** 10.7759/cureus.57925

**Published:** 2024-04-09

**Authors:** Sofia Grigoria Athanasopoulou, Mark Cicero, Elizabeth Sanseau, Maybelle Kou, Marc Auerbach

**Affiliations:** 1 Department of Pediatrics, Section of Pediatric Emergency Medicine, Yale School of Medicine, New Haven, USA; 2 Division of Emergency Medicine, Children's Hospital of Philadelphia, Philadelphia, USA; 3 Department of Emergency Medicine, University of Virginia School of Medicine, Inova Fairfax Medical Campus, Falls Church, USA

**Keywords:** foamed, newborn resuscitation, mixed reality, simbox, emergencysimbox, paediatric resuscitation, prehospital training, ems education, neonatal resuscitation program (nrp), simulation in medical education

## Abstract

Objectives: Few studies have described the current clinical practices, adherence to guidelines, and outcomes of newborn resuscitations attended by emergency medical services (EMS). SimBox, a novel, video-augmented simulation, was used to describe the adherence of prehospital providers to Neonatal Resuscitation Program guidelines, to measure satisfaction with the simulation intervention, and to describe the self-reported improvement in knowledge, skills, and attitudes after the simulation.

Methods: A prospective observational cohort study of EMS providers was designed and conducted using SimBox, an open-access simulation platform, and facilitated by EMS educators. Clinical performance measures were collected using a five-item checklist. Simulation satisfaction measures were collected through net promoter scores. Learners’ demographics, and self-reported knowledge, skills, and attitudes were measured using a retrospective survey of 25 questions.

Results: In total, 33 facilitator and 55 learner surveys were collected across Connecticut, Colorado, and Alaska between July 2021 and September 2022. At least one deviation from clinical guidelines occurred in 22/30 (73.3%) of the sessions, with 10/30 (33.3%) teams inappropriately performing chest compressions, 5/31 (16.1%) teams not warming, drying, stimulating, and suctioning the newborn, and 7/31 (22.6%) teams not performing positive pressure ventilation correctly. Lastly, 10/30 (33.3%) teams administered an incorrect dose of dextrose-containing fluids. Very high levels of satisfaction were reported with net promoter scores of 97 and 82 out of 100 for the facilitator and learner surveys, respectively. Finally, all 55/55 (100%) of the learners strongly or somewhat agreed that the simulation improved their knowledge, teamwork, communication, and psychomotor skills.

Conclusions: In this cohort of prehospital providers, clinical management decisions during a newborn resuscitation simulation often deviated from the gold-standard, newborn resuscitation guidelines. Free, online, open-access simulation resources like SimBox can be used to identify and measure practice deviations from standardized resuscitation protocols in the prehospital setting.

## Introduction

Out-of-hospital newborn deliveries are associated with increased infant and maternal morbidity and mortality. In 2021, 51,642 home births occurred in the United States, accounting for 1.41% of all births and the highest level since 1990 [1]. Neonatal mortality for unplanned home births is estimated to be 27.98 per 10,000 live births, compared with 3.27 for hospital midwife-attended births, and 13.66 for all planned home births [2].

Notably, out-of-hospital newborn deliveries are more common in rural areas with longer travel times to birthing centers [3]. The ongoing closures of maternity centers in the United States are creating “maternity care deserts” [4], which are disproportionately affecting rural areas. Fewer than 50% of rural women have access to perinatal services within a 30-mile drive from their home and more than 10% of rural women drive 100 miles or more for those services; these conditions are more pronounced in the Black and Hispanic communities and disproportionately affect low-income women [5].

The Neonatal Resuscitation Program (NRP) course, created by the American Academy of Pediatrics (AAP) and American Heart Association (AHA) in 1987, is a requirement of all providers caring for newborns in the delivery room in most institutions in the United States. NRP has contributed to an overall decrease in neonatal mortality [6], from almost 20 deaths per 1000 live births in the 1960s to a current rate of approximately four deaths per 1000 live births [7]. Similarly, the Helping Babies Breathe curriculum, which was developed for resource-limited settings and focuses on the immediate application of basic steps such as drying, stimulation, and suctioning of newborns, has been associated with a 47% reduction in early neonatal mortality within 24 hours of life and a 24% reduction in stillbirths [8].

Even though emergency medical services (EMS) often attend out-of-hospital newborn deliveries, there are no standardized national training programs or course certification requirements about newborn resuscitation for prehospital providers in the United States. Furthermore, variations exist in the training and educational requirements, as well as protocols for care and scope of practice across different EMS agencies nationally. Although practice deviation from evidence-based guidelines is associated with increased patient morbidity and mortality [9], few studies have described the current practice, adherence to guidelines, and outcomes of EMS-attended newborn resuscitations [10].

The SimBox [11-12] is a free, open-access, simulation-based resource that has been successfully used to improve the self-efficacy of prehospital providers caring for acutely ill children [13]. Each SimBox case is comprised of a video-based simulated case, accompanied by a corresponding facilitator case booklet with simulation instructions and a teaching manual. Each video combines frames of a prerecorded video of a simulated patient next to a frame of a monitor with a sequence of corresponding vital signs. The case videos are available to stream via YouTube (™) and include a structured, narrated pre-brief and debrief those facilitators could opt to use in an in-situ, hybrid, or distance simulation.

For this study, we created and used a SimBox newborn resuscitation simulation for prehospital providers with two main objectives. The first objective was to describe current practice, as well as identify and measure practice deviation from current guidelines. The second objective was to measure learner and facilitator satisfaction with the simulation intervention and to improve prehospital provider self-reported knowledge, skills, and attitudes with regard to out-of-hospital newborn delivery resuscitations.

## Materials and methods

Study design

This was a prospective observational cohort study of prehospital providers taking part in an out-of-hospital newborn resuscitation simulation, facilitated by EMS educators, and was designed following the STROBE (Strengthening the Reporting of Observational Studies in Epidemiology) guidelines [14] for research studies using simulation. The study was reviewed by the local institutional review board (IRB) and deemed exempt (approval number: 2000022435 by the Yale University Institutional Review Board), as a standard educational intervention. The first objective was to measure the learners’ performance and the second objective was to assess satisfaction with the educational intervention as well as the changes in learners’ knowledge, skills, and attitudes.

Setting

The simulations took place between July 2021 and September 2022, at the learning centers or firehouses of 10 EMS agencies in Connecticut, Colorado, and Alaska. Nine out of 10 agencies were municipal/ paid EMS agencies (West Haven Fire, New Haven Fire, North Haven Fire, West Shore Fire, Branford Fire, Stratford EMS, Upper Pine River Fire Protection District in Colorado, and Central Emergency Services Fire Department in Alaska), and one was commercial/ paid (American Medical Response [AMR] New Haven).

Participants

The learners were prehospital clinicians participating in their local agencies’ pediatric in-person educational sessions. Eligible participants were paramedics, emergency medical technicians (EMTs), and emergency medical responders (EMRs), with variable years of experience in the field and variable levels of experience with pediatric resuscitations. All participants had either Advanced Life Support (ALS) or Basic Life Support (BLS) certifications; however, Pediatric Advanced Life Support (PALS) or Neonatal Resuscitation Protocol (NRP) certification was not a requirement for participation in the simulations. Between two and five learners participated in each simulation, which was facilitated by one to two volunteer EMS educators.

The EMS educators were either pediatric emergency medicine physicians with experience in facilitating simulations, or paramedics and emergency medical technicians who have an active role in the respective agency’s educational activities. All educators who were paramedics or emergency medical technicians observed at least one SimBox simulation prior to co-facilitating their own. All the educators were offered a SimBox train-the-trainer session prior to facilitating the simulation and had access to the SimBox facilitator guide [15].

Variables: outcome measures

Our primary clinical performance outcome measures were assessed via online facilitator surveys. They included the following: whether the team started with drying, warming, stimulating and suctioning the newborn, whether they performed positive pressure ventilation and verbalized the “MR SOPA” mnemonic (Mask adjustment, Reposition airway, Suction mouth and nose, Open mouth, Pressure increase, Advanced airway), whether they identified and correctly treated neonatal hypoglycemia, by administering the appropriate dextrose-containing fluids, and finally, whether they performed any chest compressions (Table 1). Of note, based on the specific case scenario and the NRP algorithm, the learners should not perform any chest compressions during that case, and they should focus on optimizing the newborn's airway and breathing instead. Therefore, performing chest compressions at any point during that simulation was considered an "error", or deviation from the standard of care.

**Table 1:** Clinical performance measures

Clinical Performance Measures
Did the team start with drying, warming, stimulating, and suctioning the neonate (all completed or verbalized)?
Did the team perform positive pressure ventilation with good seal AND verbalize the “MRSOPA” mnemonic (MRSOPA: Mask adjustment, Reposition airway, Suction mouth and nose, Open mouth, Pressure increase, Advanced airway)?
Did the team check a glucose level at any time?
Did the team administer the correct volume of dextrose 10% intravenously?
Did the team perform any compressions during the case (ERROR)?

Secondary outcome measures were constructed to assess the simulation as an educational intervention via the learners’ self-reported satisfaction with the intervention and simulation efficacy. These were collected via retrospective online learners’ surveys and are fully listed on Table 2. Both the facilitators and learners were asked how likely they would recommend this intervention to a colleague on a scale of “Not at all likely” or zero, to “Extremely likely”, or 10, so that net promoter scores could be calculated.

**Table 2:** Simulation satisfaction and simulation as an educational intervention measures.

Simulation satisfaction measures and simulation as an educational intervention measures (possible answers: “Do not agree”, “Somewhat agree”, “Strongly agree”)
This session improved my level of knowledge for my next newborn delivery in the field.
This session improved my level of comfort for my next newborn delivery in the field.
This session improved my teamwork and communication skills for my next newborn delivery in the field.
This session improved my psychomotor skills for my next newborn delivery in the field.
The prebriefing increased my confidence.
The prebriefing was beneficial to my learning.
The debriefing contributed to my learning.
The debriefing allowed me to verbalize my feelings before focusing on the scenario.
The debriefing was valuable in helping me improve my clinical judgment.
The debriefing provided opportunities to self-reflect on my performance during simulation.
The debriefing was a constructive evaluation of the simulation.
After the simulation, I have a better understanding of the newborn resuscitation algorithm.
After the simulation, I am more comfortable managing a newborns’ airway and breathing.
After the simulation, I am more comfortable applying positive pressure ventilation in a newborn.
After the simulation, I know when to perform cardiopulmonary resuscitation (CPR) in a newborn delivery.
After the simulation, I am better prepared to respond to a delivery in the field.
After the simulation, I am more comfortable communicating with the parents.
After the simulation, I am more comfortable communicating with my colleagues.
On a scale of “Not at all likely” =0, to “Extremely likely” =10, how likely are you to recommend this intervention to a colleague?

Variables: demographic data

Learner data collected included: the learners’ role (paramedic or EMT), the name of their agency and the state in which it was located, the number of newborn deliveries they had attended in their career, if they had completed the NRP course, as well as the number of newborn delivery simulations they had attended in the past five years, if any.

Facilitator data collected included: the facilitator’s role (physician, nurse, paramedic, EMT), the name of their affiliation or agency and the state in which it was located, and if at least one learner in the team had completed an NRP course in the past.

Simulation intervention

For this study, a unique SimBox case was created with the intent to measure the performance of EMS providers in the resuscitation of a newborn baby in the prehospital setting.

Prehospital providers in the US universally have access to simple mannequins and expressed the need to heighten the fidelity (or perceived realism) of the simulation using video augmentation and the option to remotely connect expert facilitators. This is what informed the decision to use the simulation method of mixed reality human simulation [16]. In mixed reality systems, the use of technologies such as video, augmented reality, or virtual reality is used in conjunction with a physical mannequin to simulate a human [17]. In the video used for the study [18], the simulated patient was a high-fidelity neonatal manikin in various stages of distress.

During each in-person session, the YouTube video was streamed on a laptop that was placed adjacent to a low-technology mannequin (without the ability to capture breathing or chest compression data) in the educational centers or firehouses of the EMS agencies. The video provided visual information about the newborn's clinical status and changes in vital sign trends. The learners used their own equipment to practice hands-on clinical skills on the actual physical mannequin, including but not limited to, suctioning, drying, and applying positive pressure ventilation. The learners' equipment included either the actual bag with supplies and medications that they use in the field, or local equipment used for simulations, with supplies and medications similar to what they would have available in the field.

The simulation case and booklet [15] content were developed in accordance with the NRP guidelines. The case booklet included an EMS educational milestone checklist, teaching content, infographics, links to resources, as well as a pre-briefing and debriefing guide. The materials were reviewed and iteratively revised by a team of pediatric emergency medicine physicians and a neonatal intensivist. Both the booklet and the video were published and made openly accessible on the SimBox website [11] in October 2020.

We adapted a performance checklist to assess neonatal resuscitation mega-code skills [19] in collaboration with a neonatal intensivist to create a shorter, not-yet validated, checklist for this study. The case stem was of a 22-year-old female without prenatal care who called EMS as she delivered her newborn baby spontaneously at home. Upon EMS team arrival, the full-term neonate was floppy and not crying. The first step of the resuscitation included drying, warming, stimulating, and suctioning the newborn. Despite that, the newborn was still not breathing, so the participants proceeded to the next step which included applying positive pressure ventilation. However, there was neither chest rise nor rise in the heart rate with positive pressure ventilation (bag-valve-mask). The next step included the application of the “MR SOPA” corrective steps to optimize the newborn’s ventilation. These interventions led to improvement of the newborn’s heart rate, oxygen level, and level of alertness. Finally, the newborn was hypoglycemic and needed intravascular access with the administration of dextrose-containing fluids.

Data sources/ statistical methods

Data were collected using an online learner and facilitator survey. The data and results were analyzed in January 2023 using Qualtrics XM software (Qualtrics International Inc., Seattle WA), Microsoft Excel version 16.75.2 (23071901) (Microsft Corp., Redmond WA), and R version 4.0.2 (2020-06-22) (R Foundation for Statistical Computing, Vienna, Austria).

## Results

Participants

A total of 55 learners and 33 facilitators completed the survey between July 2021 and September 2022 across three states in the United States: Connecticut, Colorado, and Alaska.

Descriptive data

Of the facilitators: 26/33 (78.79%) were pediatric emergency physicians, 5/33 (15.15%) were paramedics and 2/33 (6.06%) were emergency medical technicians (EMTs). Of the learners, 46/55 (83.64%) were paramedics, 8/55 (14.55%) were EMTs, and 1/55 (1.82%) were emergency medical responders (EMRs). A total of 43/55 (78.18%) of the learners’ agencies were in Connecticut and included AMR New Haven, West Haven Fire, New Haven Fire, North Haven Fire, West Shore Fire, Branford Fire, Stratford EMS, and Yale New Haven Health Sponsor Hospital. Other agencies included the Central Emergency Services in Alaska (10/55; 18.18%), and the Upper Pine River Fire Protection District in Colorado (2/55; 3.64%).

Approximately a third of the learners, 19/55 (34.55%), had never attended a delivery in the field or prehospital setting, whereas 8/55 (14.55%) had attended one, and 23/55 (41.82%) had attended between two and five deliveries. Lastly, 4/55 (7.27%) had attended between six and 10, and 1/55 (1.82%) learner had attended over 10 deliveries. The majority of the learners, 32/55 (58.18%), had never completed an NRP course. However, most learners had participated in newborn delivery simulations in the past five years, as follows: 6/55 (10.91%) participated in one, 34/55 (61.82%) in two to five, 5/55 (9.09%) in six to 10, 2/55 (3.64%) in over 10, whereas 8/55 (14.55%) did not participate in any (Table 3).

**Table 3:** Facilitator and learner demographics PEM: pediatric emergency medicine; EMTs: emergency medical technicians; EMRs: emergency medical responders; NRP: Neonatal Resuscitation Program

Demographics	Group	Counts (%)
Facilitator Role	PEM Physicians	26/33 (78.79%)
	Paramedics	5/33 (15.15%)
	EMTs	2/33 (6.06%)
Learner Role	Paramedics	46/55 (83.64%)
	EMTs	8/55 (14.55%)
	EMRs	1/55 (1.82%)
Location of the learners’ agency	Connecticut	43/55 (78.18%)
	Alaska	10/55 (18.18%)
	Colorado	2/55 (3.64%)
Learners who had completed an NRP course in the past	Yes	23/55 (41.82%)
Number of newborn deliveries attended in the field per learner	0	19/55 (34.55%)
	1	8/55 (14.55%)
	2-5	23/55 (41.82%)
	6-10	4/55 (7.27%)
	>10	1/55 (1.82%)
Number of newborn delivery simulations attended in the past 5 years per learner	0	8/55 (14.55%)
	1	6/55 (10.91%)
	2-5	34/55 (61.82%)
	6-10	5/55 (9.09%)
	>10	2/55 (3.64%)

Clinical outcome measures

**Figure 1:**
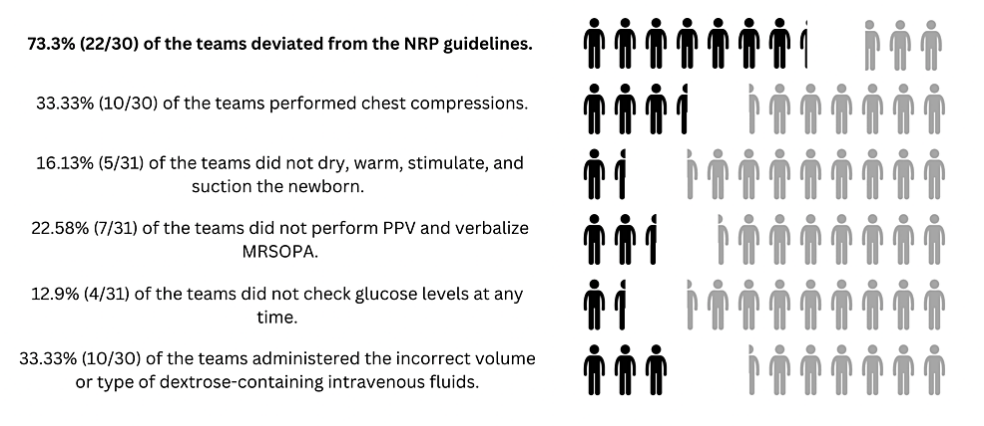
Clinical outcome measures results NRP: Neonatal Resuscitation Program, PPV: positive pressure ventilation, MRSOPA: Mask adjustment, Reposition head, Suction mouth and nose, Open mouth, Pressure increase, and Alternative airway.

At least one deviation from guidelines was identified in 22/30 (73.33%) of the teams. More specifically, a third of the teams, 10/30 (33.33%) inappropriately performed chest compressions at some point during the case, whereas 5/31 (16.13%) of the teams did not dry, warm, stimulate, and suction the newborn. Furthermore, 7/31 (22.58%) of the teams did not perform positive pressure ventilation correctly and did not verbalize the corrective steps of “MRSOPA”. Finally, even though 27/31 (87.10%) of the teams checked a glucose level at any time, only 20/30 (66.67%) administered the correct volume and type of dextrose-containing fluids (Figure 1). 

Simulation as an educational intervention measures

**Figure 2:**
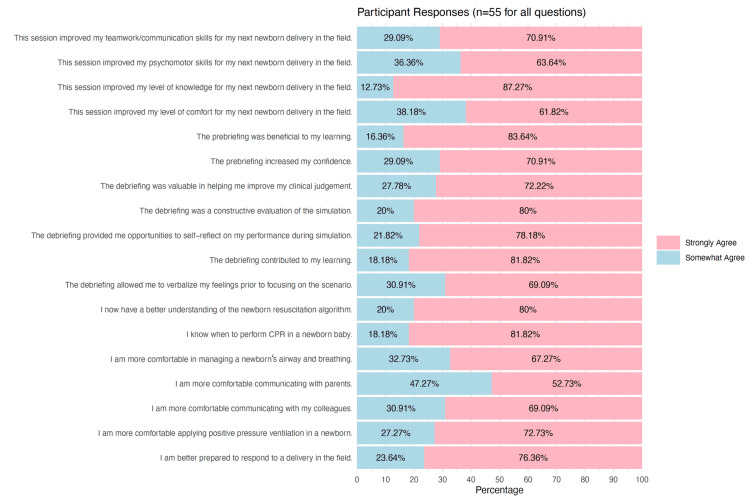
Simulation as an educational intervention results: learners’ self-reported satisfaction and self-efficacy Of note, no learner stated "Do not agree" in any of the questions.

The net promoter score was 97% and 81.8% for the facilitator and learner survey, respectively. All of the learners (55/55, 100%) strongly or somewhat agreed that the session improved their level of knowledge, teamwork, communication, and psychomotor skills, as well as attitudes with regard to attending a newborn resuscitation in the field (Figure 2).

## Discussion

Key results

In this cohort of prehospital providers participating in a newborn resuscitation simulation, an adapted performance checklist demonstrated multiple deviations from the NRP guidelines. Our study demonstrated overall very high participant satisfaction and high self-reported perceived impact of the simulation on the learners’ knowledge, skill, and attitudes.

Previous studies have identified practice deviation from NRP guidelines by prehospital providers, especially with regard to drying, warming, stimulating, and suctioning the baby [20]. These practice deviations are not only frequent, but also have the potential for mild and even severe harm [21]. Similar deviations were identified during our study, which also included a high incidence of chest compressions performed when they should not have been. Of note, the neonatal resuscitation guidelines differ significantly from adult and pediatric protocols, such as the PALS. An important difference is that the mainstay of management of a newborn is the optimization of their airway and breathing, and extremely rarely will chest compressions be required. Indeed, the Helping Babies Breathe curriculum does not teach chest compressions or the use of epinephrine, as it is very infrequently needed in newborn resuscitations. In contrast to NRP, pediatric guidelines dictate that if a patient is not breathing and is bradycardic with a heart rate of less than 60 beats per minute, chest compressions need to be initiated promptly. The observed practices during our simulation study could be reflective of the learners’ low familiarity with the neonatal guidelines and the lack of standardized training in newborn resuscitation.

A high incidence of serious patient safety events during high-risk neonatal calls in the prehospital setting has been reported, especially with regard to medication administration, resuscitation, and procedures [10]. In keeping with the existing literature, our study demonstrated frequent errors in the appropriate dosing of dextrose-containing fluids for neonatal hypoglycemia. Weight-based dosing poses another challenge in the care of newborn and pediatric patients: some teams utilized the Broselow tape, some used online/ mobile applications, and some teams did not calculate or verbalize a weight. Confusion was noted in the unit of measurement (kilograms versus pounds), as well as during attempts to convert from pounds to kilograms. A potential solution to this problem could be the standardization of the algorithmic/ medication aids and units of measurement used in the prehospital setting.

Our study also demonstrated very high satisfaction with the educational intervention and self-reported improvement in teamwork, communication, and psychomotor skills and attitudes after the simulation. Both the SimBox facilitator booklet and train-the-trainer sessions prioritize incorporating TeamSTEPPS (Team strategies and tools to enhance performance and patient safety) [22] in the debriefing, an evidence-based set of tools designed to improve communication and teamwork skills in simulation. Therefore, SimBox or similar open-access simulation resources can be used as adjunct medical education tools to foster education and adherence to the neonatal resuscitation protocol guidelines.

The use of the mixed reality human simulation SimBox scenario has multiple advantages. The use of the video to demonstrate the progression of the patient’s clinical status and vital signs does not require the presence of a simulation specialist, special equipment, or attendance at a simulation center, therefore providing accessibility. Novice and expert simulation facilitators alike have reported that having the pre-recorded prebrief and debrief and streamlining the vital sign monitor relieves their mental load during simulation facilitation, allowing them to focus on nuances of the simulation which ultimately enhances their debriefing. Some feel more comfortable facilitating the simulations on their own (when a partner is not available to help) and feel more adept at doing so using SimBox.

Training prehospital providers in facilitating simulations using high-quality, expert-written peer-reviewed, open-access medical education tools such as SimBox, could potentially help make practicing pediatric scenarios more accessible and frequent. That is true not only for low-frequency, high-stakes clinical scenarios such as newborn resuscitations but also for frequent, low-stakes scenarios such as asthma or bronchiolitis. This is particularly important in rural areas or areas with limited access to pediatric or simulation specialists. Making frequent, high-quality pediatric simulation training for prehospital providers accessible and sustainable can potentially help improve patient outcomes and address disparities in care.

Limitations

An important limitation of our study is the small number of learner and facilitator surveys completed. Although our study included EMS agencies from both urban and rural locations, expanding the study to more EMS agencies across different states could provide valuable information for future studies. Additionally, a few surveys were not completed in full but were included in the results. For surveys that were not completed in full, we included the answers to the questions that were answered but did not include any questions that were left unanswered in our results. Future studies can include only fully completed questionnaires or require all responses.

Furthermore, during some sessions, more than one facilitator completed the survey. This can be addressed in the future by standardizing the facilitator training and providing clear directions for completing the facilitator survey. Despite these limitations, we believe that the overarching themes identified in this study reflect existing practice deviations and patterns.

A final limitation is the potential for variability in educators’ adherence to the SimBox intervention. Although all the educators were provided a SimBox train-the-trainer session and had access to the SimBox facilitator guide, adherence to the SimBox scripting was not evaluated. For example, prompts may have been provided by educators to the learners (e.g., to check the blood sugar level of the patient). A way to address this limitation would be to collect data on deviations from the scripting/prompts of SimBox using an observer and/or video recordings. In future studies, the results of sessions with poor adherence to scripting can be excluded by the analysis. Another method to address variability in facilitation could be the use of recorded pre-brief and scripted debrief prompts within the video. Future studies can further assess the facilitator and learner experience by using the narrated prebrief and debrief.

## Conclusions

In this cohort of prehospital providers, deviations from newborn resuscitation guidelines were common during simulations. The simulation was a well-accepted tool by both facilitators and learners, and learners reported improved knowledge, skills, and attitudes with regard to applying the neonatal resuscitation algorithm in the prehospital setting. Free, online, open-access simulation resources such as SimBox can help identify, measure, and address practice deviations from standardized resuscitation protocols in the prehospital setting. Further studies are required to measure retention of knowledge and skills, trends in clinical practice, and patient outcomes.
